# From Soundwave to Soundscape: A Guide to Acoustic Research in Captive Animal Environments

**DOI:** 10.3389/fvets.2022.889117

**Published:** 2022-06-16

**Authors:** Fay E. Clark, Jacob C. Dunn

**Affiliations:** ^1^Behavioural Ecology Research Group, School of Life Sciences, Anglia Ruskin University, Cambridge, United Kingdom; ^2^School of Psychological Science, Faculty of Life Sciences, University of Bristol, Bristol, United Kingdom; ^3^Biological Anthropology, Department of Archaeology, University of Cambridge, Cambridge, United Kingdom; ^4^Department of Cognitive Biology, University of Vienna, Vienna, Austria

**Keywords:** animal behavior, acoustics, noise, sound, laboratory, farm, zoo

## Abstract

Sound is a complex feature of all environments, but captive animals' soundscapes (acoustic scenes) have been studied far less than those of wild animals. Furthermore, research across farms, laboratories, pet shelters, and zoos tends to focus on just one aspect of environmental sound measurement: its pressure level or intensity (in decibels). We review the state of the art of captive animal acoustic research and contrast this to the wild, highlighting new opportunities for the former to learn from the latter. We begin with a primer on sound, aimed at captive researchers and animal caregivers with an interest (rather than specific expertise) in acoustics. Then, we summarize animal acoustic research broadly split into measuring sound from animals, or their environment. We guide readers from soundwave to soundscape and through the burgeoning field of conservation technology, which offers new methods to capture multiple features of complex, gestalt soundscapes. Our review ends with suggestions for future research, and a practical guide to sound measurement in captive environments.

## 1. Introduction

**Figure 1:**
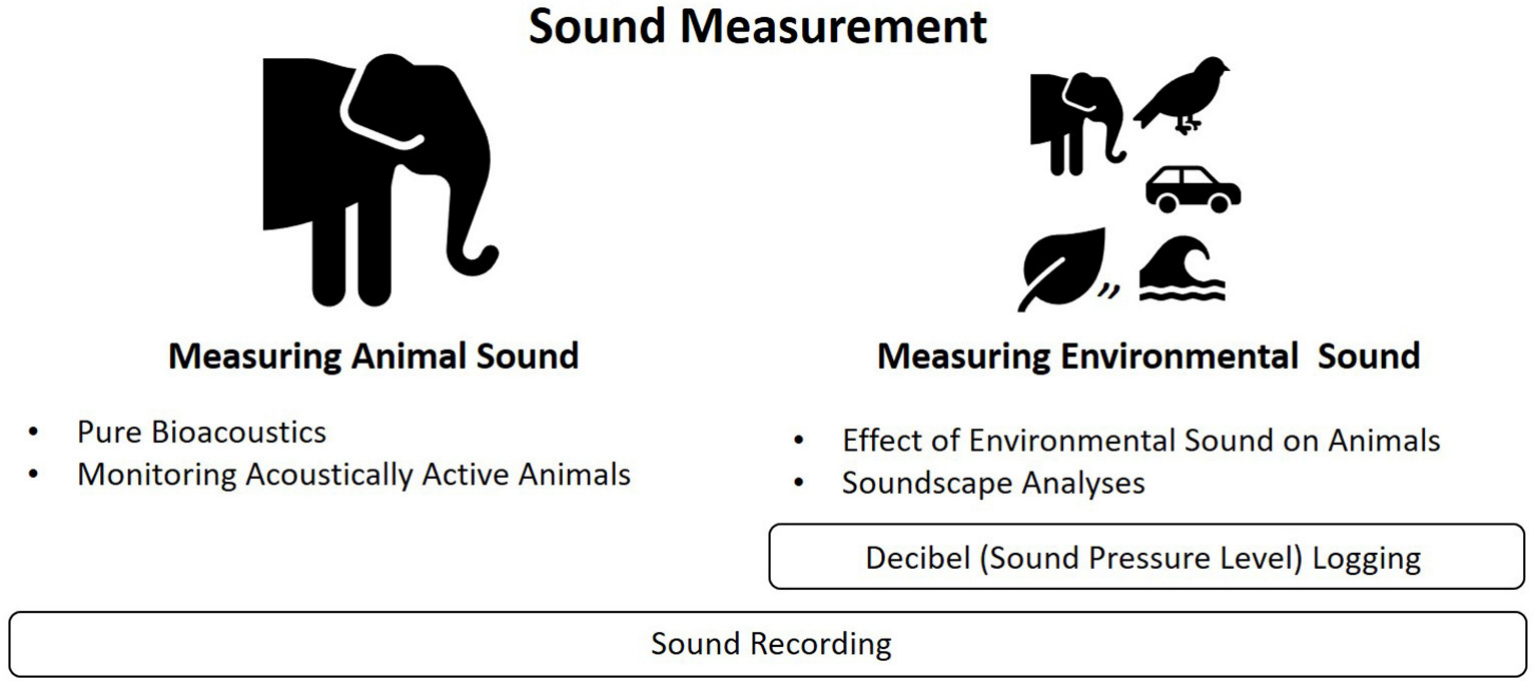
Themes of acoustic research in animals.

Sound is a complex feature of all environments and has multiple properties and features, including pressure level, frequency, and occurrence across space and time. Acoustic research on animals can be broadly split into taking sound measurements from animals themselves, or from the wider environment (Figure 1). The former includes pure bioacoustics [i.e., how animals produce, detect, discriminate, recognize and respond to sound; (1)], as well as monitoring sound to track animal abundance, distribution, health, or welfare. The latter includes research into how sound affects animal welfare, as well as into the nature of sound as an overall “soundscape”. Although most acoustic research has been carried out in wild animal populations, there is a growing movement toward acoustic research in captive animal environments (e.g., farms, laboratories, pet shelters, and zoos). This can be for direct reasons (e.g., a researcher is interested in the effects of the environment) or indirect reasons (e.g., because captive animals are easier to study than their wild counterparts).

Acoustic research in captive environments has been challenging to date. Enclosures can affect the properties of sound and limit how animals are able to spatially respond (2, 3). An animal's response to sound cannot be determined without also measuring the sound in a meaningful way, but it appears much captive literature has not achieved this balance (4). The literature shows a predominant focus on measuring maximum sound pressure levels (in decibels, dB) of environmental sound in captivity, but decibels are only a small part of the complexity of sound. Another issue is that the captive literature is disjointed and inconsistent, often based on small-*n* case studies and lacking explicit methodological detail. The replication crisis is notable across several fields of animal research (5) and acoustics follows this trend (6–9). We also recognize that the breadth and technicality of acoustics literature can be overwhelming to those new to the field (6) and literary resources for practical sound measurement in captive environments are lacking.

This review is targeted toward captive animal caregivers and researchers who have an interest, rather than expertise in, acoustics. Its purpose is to summarize acoustic research on captive animals and contrast it to research on wildlife, for which there is already a literature base [e.g., acoustic monitoring (4, 6, 10); effects of noise on wildlife (11, 12)]. In doing so, we pinpoint specific field approaches which could potentially transfer to captive environments. Our review aims to cover the whole captive spectrum and diversity of acoustic methods used to date. We focus on the “home” enclosures of animals (rather than controlled sound booth experiments), and crucially we focus on acoustic *methods* rather than the ensuing study findings. We begin with accessible background information on sound and animal hearing, before moving into a review of research. We hope to produce a comprehensive, one-stop resource to encourage advances in, and better reporting of, acoustic methods in captive animal studies.

## 2. Sound: A Primer

### 2.1. What Is Sound?

To begin broadly, *sound* is produced when an object causes vibrations of the air molecules around it. These vibrations can be represented as a longitudinal pressure wave, which can pass through air, water, or solids. *Acoustics* refers to the scientific study of sound, and more specifically *bioacoustics* is the scientific field concerning how animals produce, detect, discriminate, recognize and respond to sound (1). In our review, we use acoustics as an umbrella term to encompass bioacoustics and sound measurements from the wider environment. We find this distinction useful because a large proportion of the research we will review has not taken measurements from the animal and therefore does not strictly fit the definition of bioacoustics.

Sound is a complex mixture of pressure variations that change in numerous ways over space and time. Researchers have sought to characterize sound using a handful of metrics (4), but the two most common are *amplitude* and *frequency*. The amplitude of a sound wave relates to the number of air molecules that are displaced by the vibration (sound pressure level) and thus the perceived loudness of the sound. Change in sound amplitude is usually measured using decibels (dB), which is a general measure of the ratio between two quantities developed by Alexander Graham Bell. It has most famously been applied to sound pressure levels (dBSPL), although it is also used to quantify several other physical properties (e.g., dBV for voltage magnitude). While the decibel is the gold-standard measure of sound amplitude, there are several important challenges to consider when using it. First, decibels are measured on a log scale, so for example the difference between 0 and 10 dB is a x10 increase in sound intensity, and the difference between 0 and 20 decibels is a x100 increase in sound intensity. Decibel comparison across different studies can be difficult because the decibel is not a true unit. Unlike a meter or second, it does not have a definable size. Instead, it is used to express a level relative to a reference value [1 dB is equivalent to a pressure of 20μ Pa in the air or 1μ Pa in water; (13)]. Decibel levels are therefore meaningless without accompanying reference information on how they were measured (e.g., the pressure level and distance from the sound source). Another challenge is that, because of the log scale, two different sounds occurring at the same time cannot simply be added together to find the total decibel level. The other commonly used sound metric, frequency, is the number of vibrations (back and forth movement) of molecules per second, measured in Hertz (Hz).

### 2.2. Categorizing Sound

**Figure 2:**
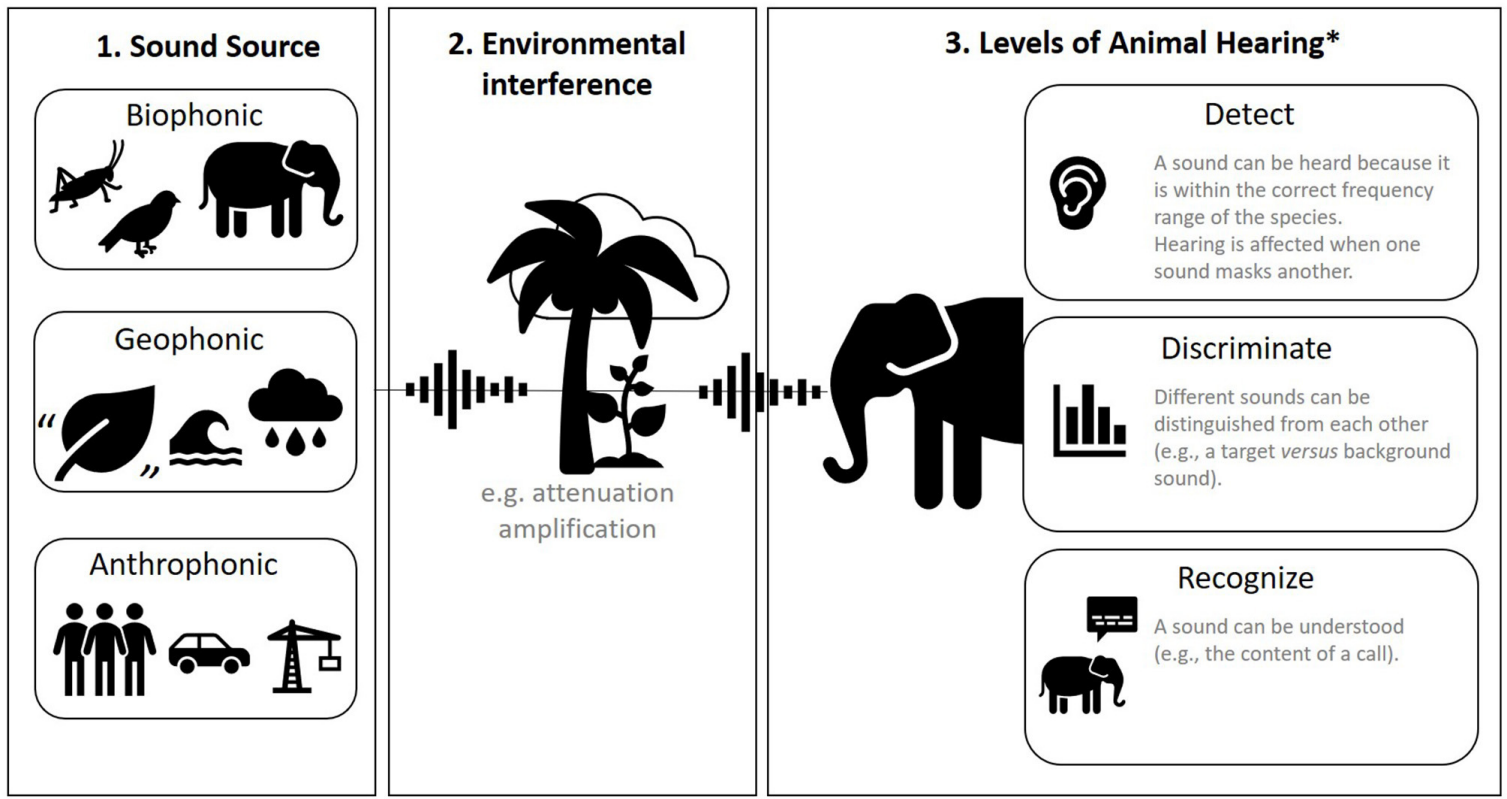
A summary of sound. Panel 1: three sources of sound. Panel 2: environmental interference with sound. Panel 3: how animals hear. ^*^Based on (14).

Sound can be categorized in many ways, but in animal research, the most common division is between *biophonic, geophonic*, and *anthrophonic* sound (Figure 2, panel 1). Biophonic sound is produced by a non-human animal, including its vocalizations and any other inadvertent sound like rustling or wing-flaps (15). Geophonic sound also comes from nature but is produced by non-living processes like wind, water, and thunder. Anthrophonic sound is any sound produced by humans, including human speech, footfall, machinery, vehicles, sound bouncing off built surfaces, etc. These categorizations can be useful because they are often discernible aurally (by ear). However, in captivity, these categorizations can become blurred because many aspects of the environment (enclosure) are man-made.

### 2.3. Noise and Environmental Interference

*Noise* is commonly referred to in the bioacoustics literature as “unwanted sound” (16). Similarly, noise has been defined as sound that serves no function to the listener (6), or sound that is unpleasant, damages hearing or hinders detection of another sound of interest (3). For simplicity, we use “sound” as an umbrella term for both wanted and unwanted sound.

Another useful consideration is how prominent or focused a sound is within space. Background sound (also called ambient sound) describes all sound other than the sound of interest. *Masking* is the process by which one sound interferes with an animals' detection of another sound; for example, when background sound covers up a sound that is of interest or importance to the animal [(17); Figure 2, panel 2]. The environment is an integral part of what type of sound is generated (biophonic, geophonic, anthrophonic), but also how sound is perceived by animals. Environments are very rarely homogenous, and the nature of sound changes spatially with varying substrates, humidity, and air pressure. For example, different types of rock, vegetation, or animals themselves can absorb or reflect sound in different ways. The inverse square law of sound attenuation (for every doubling of distance from the sound source, sound pressure level decreases by 6 dB) does not exactly hold in “normal” heterogeneous environments, but it remains a good rule of thumb (18).

### 2.4. Sound Visualization

**Figure 3:**
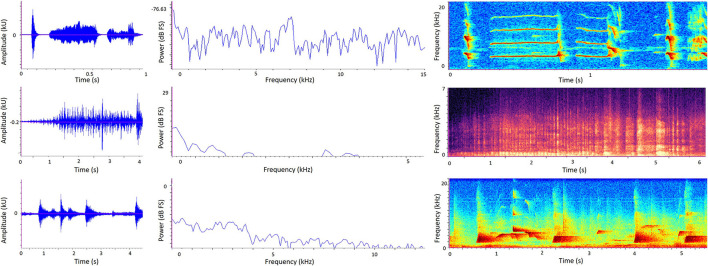
Sound visualization graphs generated from sound recordings in zoos. Left to right: Oscillogram, Power spectrum, Spectrogram. Top: Golden lion tamarin (*Leontopithecus rosalia*) calls in the absence of background sound. Middle: A zoo soundscape containing biophonic (bird calls) and anthrophonic (human speech) elements. Bottom: Western lowland gorilla (*Gorilla gorilla gorilla*) calls in the absence of background sound. Note differently scaled axes to best suit different species and environment. In the spectrograms (right), color intensity represents amplitude. Here, darker colors represent lower amplitude and lighter colors represent higher amplitude.

Three types of graph allow us to visualize the temporal and/or spectral characteristics of sound: *oscillograms, power spectra*, and *spectrograms* (Figure 3). An oscillogram (Figure 3, left) is a 2D graph showing changes in a sound signal's amplitude over time, with amplitude on the *y*-axis and time on the *x*-axis. A researcher may replay a recording and observe the oscillogram at the same time to learn what certain animal vocalizations or other discrete sound events look like; then they can use oscillograms to visually detect the occurrence of particular sounds in future recordings. Figure 3 (left) shows how different sounds can quickly be discriminated visually from oscillograms alone. Oscillograms are also useful for calculating the total amplitude of a recording.

A power spectrum (Figure 3, middle) is a 2D graph showing how a sound metric such as sound pressure level (dB) on the *y*-axis varies with frequency (Hz) on the *x*-axis. By looking at a power spectrum of a recording, a researcher can therefore deduce how much sound energy (or power) there is at different frequencies of the sound signal and therefore whether a sound is relatively richer in lower or higher frequencies. More specifically, the graph shows the average power in each frequency band, plotted against the middle value of the frequency band. When considering very wide frequency ranges, it is useful to divide them into equally-sized bands called *octaves*. Sound frequency doubles with each octave, so for example there is one octave between 1,000 and 2,000Hz. One technique to produce a power spectrum is using the *Fourier transformation*, which produces a set of equal frequency bands and tells us how much energy (power) is contained within each of these. Power spectrums are useful when a researcher is interested in a summary of frequency composition over a given time period, rather than how frequency changes over time.

A spectrogram (Figure 3, right) is a visualization of a sound recording in three dimensions: frequency (Hz) on the *y*-axis, time on the *x*-axis, and amplitude represented by color intensity. Warmer or darker colors typically reflect higher amplitudes, although this may sometimes be reversed so it is important to refer to the specific key provided with each spectrogram. Frequency (on the *y*-axis) is split into frequency bands (also known as bins). For example, a researcher might set up a spectrogram to show a maximum frequency of 7,000Hz and each bin is 20Hz, leading to 350 different frequency bands shown on the spectrogram. Similar to oscillograms, the time element of spectrograms allows researchers to view the temporal pattern of a sound relatively quickly by eye, and therefore visually detect specific sounds from a recording. But while spectrograms look visually attractive, they only express relative variations in amplitude rather than known sound pressure levels, and therefore it is challenging to compare separate recordings using spectrograms alone (19).

Several examples of acoustic software packages that can be used plot graphs from digital sound recordings are listed in Section 6.4. Oscillograms are produced directly from digital sound recordings and do not require any post-recording processing. In contrast, power spectra and spectrograms require post-recording processing and are therefore more complicated to plot. Beyond visual inspection, oscillograms, power spectra, and spectrograms can also be used to extract acoustic measurements which can then be used to calculate acoustic indices (Section 4.2).

## 3. Acoustic Research 1: Measuring Animal Sounds

### 3.1. Bioacoustic Research

We will only briefly cover fundamental studies of animal hearing in this section; they are undertaken using very specialist facilities and equipment and are therefore of little relevance to our target audience. Also, we do not cover animal cognition research where researchers have used sound as a test stimulus but are not interested in the sound *per se*. For example, domestic pig *Sus scrofa* learning has been investigated using acoustic tones as a cue (20).

#### 3.1.1. Animal Hearing

Auditory systems differ vastly across the animal kingdom (21, 22), from external mobile pinnae (“ears”) and internal ear canals to vibration-sensing bones and sensory bristles (21) to detect sound (Figure 2, panel 3). It is therefore important to perform background research on the auditory system of your study taxa or species [for example, within marine mammals there are five functionally different auditory groups; (23)]. Animals may also have certain hearing behaviors related to the orientation of the head and body, or the production of echolocation bursts in the case of bats and toothed whales (21). To complicate things further, the auditory sense is intrinsically linked to other senses; many species switch from hearing to sight or other sensory modalities when distracting sound impedes their ability to discriminate or recognize important sound (Figure 2, panel 3) (24).

A major challenge in bioacoustics is to measure sound in a meaningful way—to reflect what animals can hear, rather than what humans can hear. Specialized auditory tests have been performed on several species and give a baseline indication of what sound different taxa can hear (21). Broadly speaking, there is a negative relationship between body size and hearing capacity (highest audible frequency) (16). However, for many species, the specifics of their hearing capabilities remain unknown. Behavioral hearing tests involve playing a pure tone and training the animal to respond whenever they can hear it; the tone is gradually reduced in intensity and frequency until the animal no longer responds. However, behavioral hearing tests are biased toward animals that can be trained easily and have thus proved difficult for some taxa, e.g., herptiles (25). Alternatively, neural responses to sound can be measured using a technique called the auditory brainstem response (ABR). For this, a short pure tone or click is played (ideally in a sound-controlled booth), and electrical activity is recorded from electrodes on the animal's skin (26). Auditory brainstem responses can be conducted much faster than behavioral tests and do not require training, but it should be noted animals may respond neurologically to sound they cannot physically hear (25). An *audiogram* is a graphical representation of how well an animal can hear a sound at different frequencies, plotting decibels on the vertical *y*-axis against frequency on the horizontal *x*-axis. Audiograms are useful starting points to guide research, alongside the power spectrum of a particular sound (Section 2.4), taking into account what sound a species should be capable of hearing. However, species-level audiograms are not representative of normal hearing in “noisy” environments and also do not take into account individual variations, such as age-dependent hearing loss or damage (16, 17).

Auditory (hearing) ranges span several orders of magnitude across the animal kingdom (4), so it is not surprising bioacoustics is a vast field with distinct bodies of literature for various clades, particularly primates, bats, fish, and birds (27–29). The human ear best detects sound frequencies between 20Hz and 20 kHz, otherwise known as our audible range sounds. Sound above this frequency range is called *ultrasound*, including the echolocation signals of toothed whales and bats, and sound produced by many insects and amphibians. Sound below this frequency range is called *infrasound* and includes the vocalizations of large ungulates, pigeons, and some fish (30). Because ultrasound and infrasound are imperceptible to the human ear, they require specialist equipment for us to detect. It is important to remember ultrasound and infrasound are not only produced by animals, they are commonly produced by the environment in the form of thunder and waves, and artificially by HVAC (i.e., heating, ventilation, and air conditioning units) and other machinery (7). The *vibrations* we (and other animals) can feel as movements are often associated with low-frequency noise (31). While there is a high overlap in the hearing ranges of common laboratory species and humans (thus leading to a relatively high interest in sound we can collectively hear), there are large differences in upper and lower frequency hearing ranges across species (25). Many laboratory mammals have higher frequency hearing than humans; for example, the house mouse (*Mus musculus musculus*) can hear two octaves (frequency bands) higher than us but has poorer low-frequency hearing (25).

#### 3.1.2. Animal Sound Production

Animals purposely produce sound for many reasons, such as to communicate with conspecifics (mate attraction, territory defense, alarm calls), detect predators, forage, and navigate. The myriad ways animals produce sound, e.g., actively using specialized vocal anatomy or passively by locomoting, are reviewed elsewhere [see, (21), (30)]. We consider the production of incidental sound by animals, such as sound created by feeding, in Section 3.2.1.

#### 3.1.3. Sound Detection, Discrimination, and Recognition

A great deal of research has been undertaken on the detection, discrimination and recognition of vocalizations (Figure 2) in a range of species and contexts to understand their function [reviewed by (32)]. Once a researcher has determined the typical vocal variation or “feature space” of a species as its baseline, they can proceed to manipulate various variables affecting vocalization (9). This type of bioacoustics research may require recording spontaneous, naturally produced animal sound (Section 6.2). Or, you may need to artificially produce a sound so that it can be experimentally controlled. *Playback* experiments involve broadcasting natural or synthetic stimuli and recording the response of animals to these stimuli. Playback stimuli can be in any modality (visual, acoustic, etc.) but are most commonly associated with acoustic stimuli (33, 34). Playbacks can be used to answer many research questions relating to sound. For example, to investigate whether animals can perceive sound of various amplitude and frequencies [e.g., (35), (36)], discriminate between two or more sounds (37), recognize individuals or groups by their sound [e.g., (38)], or whether cognitive or behavioral performance is affected by the presence of sound [e.g., (39)].

### 3.2. Acoustic Monitoring

Animal sound can be used as a calling card by researchers, to detect and monitor individuals or groups across space and time. Acoustic recordings can be used to survey animal presence or abundance, behavioral category, or various characteristics such as age or sex.

#### 3.2.1. Animal Presence, Identity, and Behavior

There is a rich literature on acoustic monitoring of wildlife [reviewed by (10)]. Acoustic monitoring of wild animal presence, abundance and distribution are vital when visual monitoring is simply not possible (e.g., due to inaccessible terrain or harsh climate, or because species are rare, cryptic, or travel over hundreds or thousands of miles). Acoustic monitoring has received less attention in captivity than in the wild, due to the fact that captive environments do not have the aforementioned monitoring challenges. However, automated acoustic monitoring has immense value in intensive farm environments; facilities housing many thousands of animals under one roof are difficult to observe and have poor air quality for humans (40).

Acoustic monitoring is increasingly used in intensive farm and laboratory environments to track the identities and activities (rather than presence/absence) of animals when behavioral observation is difficult. In the laboratory, the ultrasonic vocalizations of house mice have been used to infer levels of social and sexual behaviors which would be very time-consuming to observe in person. In fisheries, acoustic monitoring has been used to detect incidental sound associated with mating, spawning, and feeding in fish and crustaceans (41, 42). Several studies have used sound (more specifically the sound of jaw movements or pecking) as a proxy for feeding in farmed ungulates and chickens [e.g., (43–46)], allowing farmers to monitor feed intake and general behavior patterns. The sex and genetic strain of intensively reared (broiler) chicks have been rapidly identified through their vocalizations, compared to very time-consuming and costly visual or genetic methods (47). The authors (47) found that the second formant, which is a specific acoustic measurement related to how the sound wave resonates, could reliably be used to identify chick sex and strain.

In practical terms, animal vocalizations need to be detectable against environmental background sound. It is possible to aurally detect vocalizations from sound recordings or to visually detect them on a spectrogram. For example, sound from a pod of zoo-housed killer whales (*Orcinus orca*) was collected using a hydrophone and then examined using spectrograms [Section 2.4, (48)]. More discrete sound produced by different individuals can be recorded using radio collars with onboard microphones, for example on African elephants (*Loxodonta africana*) (49) and laboratory-housed common marmosets (*Callithrix jacchus*) (50) but collars not practical for many species or contexts. Instead, the ability to detect and classify animal vocalizations within large groups has developed considerably with advancements in computer technology. It is now possible to use computer algorithms to pinpoint and only record vocalizations of interest, rather than recording hours of indiscriminate sound which must then be sifted manually (10). Furthermore, artificial intelligence can be used to classify vocalizations. For example, farm-housed goats (*Capra hircus*) vocalizations were categorized by individual identity, group membership, and age using a specialized computer program designed to mimic the decision-making process of the human brain (thus called an artificial neural network) (51).

Because decibels are a measure of sound energy, decibel level can also be used as a proxy for the animal's distance from the sound logger, taking into account the aforementioned differential absorption and reflection of sound due to physical features in the environment (Section 2.3). Interestingly, researchers recently developed a method to detect and discriminate wild elephant presence and behavior based on seismic data (i.e., ground vibrations) generated by the animals, providing another perspective to sound measurement (52).

#### 3.2.2. Animal Health and Welfare

The uptake of acoustics in captive animal health and welfare has been slow, despite evidence that animal vocalizations can reliably indicate emotional and physiological states (40, 53). Research in this area can be divided into the diagnosis of: (1) physical health problems; and (2) emotion as a welfare indicator.

The ability to quickly and reliably detect disease in intensively-reared animals has been well-studied because it has high commercial value. Sound-based precision farming techniques are appealing because they can be used when visibility is poor (e.g., in high animal densities, at nighttime), are non-invasive, and are robust against temperature changes (54). Most research in this area has been directed toward the detection of respiratory disease, for example in chickens (55, 56), cattle (57), and pigs (54, 58). The first step is to detect the presence of any vocalization and the second is to classify the type (such as the characteristic “rale” in chickens or “cough” in ungulates), thus leading to a disease diagnosis. For example, researchers extracted 23 acoustic features from recordings of chicken vocalizations and used discriminant function analysis, which is a multivariate statistical test of differences between groups, to identify the five best acoustic features for detecting disease (56). The authors then used an artificial neural network (Section 3.2) to detect healthy and unhealthy chickens from these acoustic features. Vocalization can also indicate reproductive health; whistle production in a zoo-housed female slow loris (*Nycticebus* sp.) reliably corresponded to estrus state (59). The authors used a method called pulse train analysis to automatically count the number of distinct calls in recordings. Estrus detection through vocalization has also been performed in farmed animals (60, 61).

Research on the connection between vocalization, emotional state, and captive animal welfare typically involves recording vocalizations under conditions of known “valence” (i.e., what we as humans believe are relatively positive or negative conditions for animals), and validating these against other welfare indicators such as behavior or heart rate (53, 62). Negative emotional states have been indicated by the vocalizations of chickens (63), goats, pigs (64, 65) and horses (66). Positive vocalizations in the form of contented “murmurs” have been postulated for cattle (67), but remain relatively understudied (62). Outside the farm, there has been considerably less work on bioacoustic welfare assessment (68, 69), presumably due to a lack of commercial value. Several studies point toward a relationship between emotional state and some characteristic(s) of vocalization, for example by calls becoming more intense or more irregular. Researchers recorded the barking of dogs in different emotional contexts and revealed barks could be classified by their emotional context, even within individual dogs (70). Similarly, reliable differences in laboratory common marmoset vocalizations were detected in situations of positive, neutral, and negative affective state, showing that recording vocalizations in a group environment is more naturalistic than lone testing (50). The “rumble” vocalizations of African elephants in a zoo during times of low and high social interaction (taken to infer relatively negative and positive emotional context, respectively) were compared, finding differences in amplitude, frequency, and duration between the two contexts. However, there was stronger evidence for vocalization indicating the intensity of emotion rather than whether it was relatively negative or positive (71, 72). “Non-linear phenomena” caused by irregular vibrations of the vocal anatomy are thought to be indicative of high emotional arousal, as identified on the spectrograms of infant giant pandas [*Ailuropoda melanoleuca;* (73)]. A study compared the barks of healthy and unhealthy dogs (the latter being housed in a veterinary clinic), calculating the harmonic-to-noise ratio (which compares regular to irregular vibrations in the call) showing healthy dogs have more regular harmonics (74). “Shimmers” and “jitters” (fluctuations in amplitude and frequency, respectively) are used to infer levels of negative stress or anxiety in human speech (75), and have also been applied to zoo-housed African elephants showing broadly similar findings (76).

For practical application, welfare-indicative vocalizations can be used to create a real-time captive animal welfare monitoring system (62), but this is rare outside of highly commercial farm environments. Beluga whales (*Delphinapterus leucas*) were less vocal several weeks after being transported to a new zoo enclosure, based on recordings made several times per day or per week (77). Rather than monitor vocalization, a long-term (i.e., over several years) behavior and welfare monitoring program at one zoo took into account daily decibel levels, thus acting as an early warning system during events or construction (78). Recently, an automated, real-time whistle (i.e., abnormal vocalization indicative of distress) detection system was devised for bottlenose dolphins (*Tursiops truncatus*) housed at a research facility (68). This allowed continuous monitoring from a hydrophone array on the floor of the enclosure and saved researchers over 6 days of manual labor per month processing acoustic data (68). Other reports of acoustic monitoring in captive wildlife have been short-term e.g., for several days or weeks but often over limited hours [e.g., galagos, *Galago* spp., (79)], rather than having permanent systems in place. Time-restricted acoustic monitoring could underestimate vocal activity, and miss rare and/or sporadic sounds with high biological significance.

## 4. Acoustic Research 2: Measuring Environmental Sounds

### 4.1. Effect of Sound on Animal Behavior and Welfare

The research we discussed in Section 3 relied on measuring sound produced by acoustically active animals. In this section, we turn our attention toward measuring sound from the environment. This includes biophonic, geophonic, and anthrophonic sound (Figure 2). As stated before, we focus on measuring sound as an independent variable rather than reviewing evidence for the effect of sound on animal behavior, welfare, or auditory system damage [instead see (80, 81)].

#### 4.1.1. Background Sound (Noise)

Captive animal facilities vary greatly, but sources of background sound can intuitively be categorized into: (1) permanent heating, ventilation, and air conditioning (HVAC) systems, and “life support” water management systems in aquaria; (2) temporary equipment for cleaning, gardening, etc., and public announcement and music systems; (3) human speech and footfall; and (4) sounds produced by animals. In addition, substrates inside animal enclosures and the surrounding area will affect sound (Section 2.3), so it is important to take sound measurements wherever animals will be living (4). The *active acoustic space* is the distance an animal can detect or produce sound (82) and will most likely be derived from pure bioacoustic research. This active space is artificially restricted in captivity, meaning that sound detection and production in captivity can be very different from an animal's evolved capabilities. *Reverberation* (the persistence of sound due to the reflection of sound waves from non-absorbent materials) is problematic in enclosures with hard and smooth surfaces (2), which are regularly encountered in captivity.

A branch of bioacoustics research that has relevance to our discussion of background sound has examined the effect of background sound on acoustic communication (22, 83). A phenomenon called the Lombard Effect exists, whereby animals increase the amplitude of their vocalization in response to an increase in background noise (84). The Lombard effect has been found in a wide range of vertebrates in both wild and captive environments [reviewed by (85)]. For example, the amplitude of vocalizations from common marmosets was positively correlated to background white noise. Animals may also restrict their calls to periods of silence; in other words, changing the timing of vocalizations to avoid them being masked by other sound in the environment (86).

The effect of laboratory HVAC sound has been investigated (87), with increased interest over the past two decades due to the potential negative impact of sound on the validity of laboratory animal models (88–90). Several studies have continuously monitored decibel levels inside laboratory animal housing (the cages themselves or communal rooms), focusing on peak and average decibel levels and attributing peak levels to cleaning equipment or worker activity [e.g., (91–93)]. Given taxonomic differences in hearing, it is advisable to record decibels at both low and high frequencies if undertaking multi-species research (91, 93). Reporting how often decibel levels exceed an arbitrary threshold [e.g., (91, 94)] might have some value, as long as the threshold is in some way meaningful for animals (for example based on prior research findings of hearing or responses). The effects of vibrations (i.e., non-audible, solid-borne sound) associated with high-intensity construction or animal transport have been also investigated in farms and laboratories. Vibrations inside the cages of laboratory rodents have been measured and compared to pre-existing reference ranges to extrapolate how vibrations would resonate inside the bodies of humans and rodents (95). Vibrations experienced by animals in transport vehicles have been replicated experimentally by placing animals onto a vibration machine capable of different frequencies and accelerations [poultry: (96); cattle: (97); pigs: (98)].

The consensus from research in aquatic environments (i.e., aquariums and zoo marine mammal enclosures), where sound waves travel about four times faster than in air, is that loud sound comes from a wide range of sources including life support systems (e.g., water pumping and filtration equipment), wave machines, cleaning equipment, visitors, and amplified music or tannoys (99, 100). Hydrophone recordings and power spectra (Section 2.4) from 15 marine mammal facilities showed a large variation in ambient sound between concrete tanks and naturalistic (e.g., penned lagoon) enclosures (99). A separate study found that loud enclosure sound did not significantly overlap with the hearing thresholds of bottlenose dolphins; the life support system produced primarily low-frequency sound, whereas dolphins have high-frequency hearing (101, 102). Taking continuous sound recordings from a dolphin pool has been used demonstrate the contribution of one piece of cleaning equipment, and how dolphins whistled less when it was being used (103). Some elegant experiments on laboratory zebrafish (*Danio rerio*) have involved creating a sound pressure gradient across the tank and investigating how fish are attracted or repelled by different levels [and how placing the tank on sound-absorbing foam can minimize uncontrollable background sound from the facility; (104)].

Sound is commonly cited as an integral aspect of the “visitor effect” in zoos, but it has proved difficult to parse the effect of visitor-generated ambient noise from other connected factors, such as crowd size and behavior (105, 106). Research in zoos has focused on audible ambient sound levels (generated by zoo visitors and other sources), summarizing maximum and average decibel levels (7, 78). One study measured the sound of visitors knocking on aquarium glass, finding a typical knock was 125 dB (relative to 1μ pa in water). Decibel readers placed in visitor areas rather than enclosures themselves [e.g., (106)], can then be difficult to interpret due to sound attenuation (Section 2.3), i.e., the noise level in the visitor area may not be the same as that experienced by the animal a few meters away.

Interestingly, several studies report animal vocalizations are a major source of sound in captive environments. For example, most sound within the optimal hearing range of toothed whales (40–100 kHz) in captive facilities is generated by the whales themselves (99). A dog bark can exceed 100 dB [measured in unweighted decibels but no reference distance of the reading was given, (107)], and barks contribute significantly to the noise level of kennels (93, 107). In a cross-taxa analysis of vocal animals, researchers found that animal taxonomic groups contained species that could produce sound above 100 dB in air (20 μPa at 1m), and a few species of mammal and bird produced sound up to 125 dB (108). This causes a real challenge for animal caregivers; attempting to prevent animals from vocalizing has negative ethical and welfare connotations, but alternatively, changing the acoustics of a captive environment may be unfavorable to management.

#### 4.1.2. Sound (Noise) Events

We define a “sound event” in captive animal environments as temporary noise (i.e., sound that has no positive function or value for the animal, Section 2.3). This may include construction work, extreme weather events (storm, thunder), out-of-hours events held in zoos, or wider community events such as festivals, airshows, and fireworks. In these cases, researchers must be prepared (often at short notice) to record sound that is out of their control. This being said, it may also be possible to artificially replicate a real sound event [e.g., construction sound playback, (109)] and experimentally evaluate the effect, assuming ethical approval is granted. Like chronic background sound, sound events are important to study because manmade sound can mask biologically important signals or cues (17), and in captivity, there may be restricted opportunity for an animal to escape aversive sound.

One study compared the performance of Japanese macaques (*Macaca fuscata*), chimpanzees (*Pan troglodytes*), and Western lowland gorillas (*Gorilla gorilla gorilla*) during baseline ambient sound and an air show lasting several days (thus the authors could not control the noise condition themselves) (110). In that study, jets flying overhead were brief noise events identifiable from spectrograms (Section 2.4), but in more chronic noise events it can be difficult to ascertain exactly what characteristic or duration of noise is problematic (if at all) for animals. Construction is difficult to place as either a chronic or acute sound event; it can be fairly sporadic and unpredictable or occur regularly for several weeks or months. Evidence for the effect of construction sound in zoos has been relatively well-reported, but uses a variety of acoustic methods [e.g., (109, 111–113). In one zoo, sound contour maps for each enclosure were created, taking into account the location of speakers (playing back experimental construction sound), topography, and ear height which could then be used to help monitor actual construction sound (109). Another study (101) reported that music from an evening event in an aquarium was detectable in a nearby beluga whale tank. The authors modeled the propagation of music from the aquarium's ballroom (air) to the beluga tank (water) via an acrylic viewing window. In contrast, several other published reports of evening events in zoos (which vary greatly by duration, visitor type, music type, fireworks and/or music, as well as species studied) monitor animal responses before, during, and after events; but these events are presumed to be, rather than quantified as, noisy [e.g., (114–117)]. Researchers recently performed a multi-species comparison of the effects of concerts on zoo animal behavior, comparing pre-, post- and during-event median decibel levels and animal behavior (118).

#### 4.1.3. Sound Mitigation

There are currently no guidelines for sound thresholds for animals in various captive environments, and most studies use human occupational health standards e.g., the National Institute for Occupational Safety and Health (119) as a guide. Maximum noise levels for workplaces, according to the World Health Organization (120), are LA_eq_ = 85 dB and LC_peak_ = 135 dB. These are roughly translatable to animals' hearing ranges similar to ours (such as great apes) but not for animals with ultrasonic and infrasonic hearing. The varying hearing sensitivities of different species found in laboratories, farms, pet shelters, and zoos make determining standard thresholds within or between environments very unrealistic. In these situations, stating the *signal-to-noise ratio* threshold may be more relevant. The *signal-to-noise ratio* refers to the difference in amplitude between the sound of interest and the background sound. These amplitudes should be as different as possible so that the latter does not mask (cover up) the former. A *signal-to-noise* ratio smaller than 25 dB [based on humans and 40 bird species; (17)] can negatively impact hearing ability.

Mitigating sound can take many forms, at the source (e.g., reducing the type or characteristics of a produced sound), during transmission (e.g., how it is reflected or absorbed by the environment), or by the receiver [e.g., how the animal perceives it; (78)]. Sound mitigations such as sound-proofing will briefly be noted here. Different sound-proof barriers will either reflect or absorb sound, so if a barrier is required for research purposes this ability must be tested experimentally before use. For example, comparing the noise-reducing qualities of plastic, wood, and foam barriers for zoo enclosures can be achieved by simply measuring the reduction in decibels from one side of the barrier to the other (78). Planting more trees in an enclosure may reduce sound levels but ironically may lead to animals finding it harder to switch to visual signals (modality switching, Section 2.3.1). *Sound conditioning* refers to masking unpleasant acoustic stimuli (noise) with another sound, such as music, white noise, or a naturalistic recording (121).

In contrast to blocking or masking aversive sounds, a small branch of research has considered adding beneficial sound into the environment, which we interpret as a form of environmental enrichment. Acoustic enrichment in zoos has had very mixed methods and results, calling for more concise statements of methods to allow replication (8). Across captive environments, it is typical to find animal behavior is compared before, during, and after the addition of pre-recorded music [e.g., chickens: (122); Western lowland gorillas: (123): Psittacines: (124)]. As one example, farmed chickens have been exposed to pre-recorded machinery sound and music composed by Mozart (122). These conditions were played for different durations of time and at different dB levels, which raises interesting debate over standardizing sound conditions *vs*. making “naturally” sporadic and variable. The sound metrics of different music types have not been rigorously studied in the context of acoustic enrichment [although see two studies that reported beats per minute in studies on domestic cats and dogs, (125), (126)], so there is scope to apply a range of indices in this field (Section 4.2). An interesting sideline has been to provide sound stimuli on-demand to animals; chimpanzees and orangutans (*Pongo* spp.) have been given control over sound production either by pressing control units or by moving physical objects in the enclosure (127, 128). Sound has also been used more as a prompt to perform more naturalistic behaviors, with little consideration for the information held in the sound *per se*. Generic bird sound has been used as a cue for an African leopard (*Panthera pardus*) to explore her enclosure, but the sound itself had little real relevance to the leopard, who had only learned the connection between the sound and a food reward (129).

### 4.2. Soundscape Measurement

We now turn our attention to a theme of acoustic research increasingly used in the field, but which may have some applications for captive environments. A *soundscape* is an acoustic scene in its totality; it is defined as the “…*ensemble of ambient sound, including sound events, associated with a specific location at a particular time*” [(3), p. 693]. Viewing sound as an environment in its own right is becoming increasingly popular (130–132) and moves away from thinking of sound by its decibel level and considers many other characteristics. Furthermore, there is increasing recognition that sound is gestalt, in other words an emergent property different from the sum of its parts (133). *Soundscape ecology* is the study of the effects of the soundscape on animals, e.g., their physiological and behavioral responses (134). An *acoustic index* is a statistic used to summarize some aspect of the diversity or complexity of a sound recording (135) and is thus inherently linked to the concept of soundscapes. Broadly, soundscape indices can be divided into two classes: α acoustic indices assess the diversity (richness or complexity) of a soundscape, whereas β acoustic indices assess the level of dis/similarity between soundscapes. The overarching benefit of indices is that they reduce the enormous complexity of a soundscape into a single number, which can in turn help summarize large quantities of acoustic data. They can be used to compare one soundscape over time (e.g., by season or year), by space (e.g., vertical layers of the forest), or compare soundscapes at different sites. Acoustic indices have received a detailed review in the literature so we restrict our discussion to soundscape indices most likely to have value to captive environments. For readers interested in gaining a deeper understanding of acoustic indices for wild research, we recommend (136, 137).

#### 4.2.1. Soundscape Complexity

The most common α soundscape indices measure the biodiversity of the soundscape and were inspired by traditional biodiversity indices used in ecology (136, 137). The acoustic complexity index [ACI; (138)] is a commonly used index comparing the difference in amplitude from one time interval to the next within a narrow frequency band. Therefore, the data required to calculate an ACI can be extracted from a spectrogram divided into temporal and frequency bins [Section 2.4, (138)]. High ACI values are obtained from soundscapes with high biophony (e.g., bird and insect calls), or geophony (e.g., storms), thus it is hard to make a clear distinction between these two sound categories. Similarly, the acoustic diversity index [ADI; (139)] measures evenness across frequency bands the required data can be extracted from a spectrogram. A soundscape containing a high range of frequencies will yield a high ADI value [but so will a completely silent recording, reinforcing the importance of listening to recordings in addition to any computer analysis, (137)]. ACI has been used to reliably estimate the number of indri lemurs (*Indri indri*) participating in a chorus, which is beneficial because inspecting spectrograms was only reliable for two detecting three or fewer singers (140).

#### 4.2.2. Soundscape Naturalness

Another common application of α indices is to summarize the degree of naturally produced (*biophonic*) or manmade (*anthrophonic*) sound in a soundscape. The normalized difference soundscape index [NDSI; (131)] is used to measure the relative ratio of biophonic to anthrophonic sound, working on the assumption that these categories of sound fall within particular frequency bands. Note that geophonic sound is merged with biophonic sound because the index works to distinguish between natural vs. manmade sound. Biophonic sound tends to fall within 2–11 kHz whereas anthrophonic sound tends to fall within 1–2 kHz frequencies (131). To calculate an NDSI, an readings are taken from a power spectral density graph (Section 2.4) for anthropogenic and biophonic frequency ranges, and a ratio is calculated. A higher NDSI value indicates less anthrophony in the soundscape, but the NDSI is by no means a flawless method. The main issue is that the frequency cutoffs are artificial; animals can produce sound below 2 kHz which would be wrongly classified as anthrophony, and the sound of wind and rain can also register as low-frequency sound. Assessing the “naturalness” of animal environments has also been achieved without using soundscape indices, but requires more subjective comparisons and reliance on decibel levels. For example, amplitudes and frequencies of zebrafish soundscapes in the laboratory and five wild habitats have been compared (141). Another study found that ambient noise level in an aquarium pool was 15–25 Db higher than the wild habitat this pool was intended to simulate, due to the life support system (142).

#### 4.2.3. Soundscape Dis/Similarity

β acoustic indices are less common than α acoustic indices and are used to compare how similar or different soundscapes are across space or time. A dissimilarity index estimates the (dis)similarity in the composition of two recordings (143), and examples include the Spectral Dissimilarity (Df), Temporal Dissimilarity (Dt), and Acoustic Dissimilarity Index (D). Soundscape dis/similarity is difficult to deduce, given that several confounds such as time of day and distance between the microphone and the sound source could wrongly be interpreted as soundscape differences, and there is no universally agreed metric to estimate sound similarity or difference (136). Spectral dissimilarity (Df) for example is derived by comparing the power spectrums (Section 2.4) of different recordings, calculating differences in average Fourier-transformed data for each frequency bin. Given that current β acoustic indices have been criticized for their simplicity and are not as straightforward to calculate or interpret as α indices (136), we issue caution with their use.

## 5. Future Directions for Captive Animal Acoustic Research

To summarize our findings, within captive environments, there seems to be a relationship between the type of acoustic research (Figure 1) and the commercial value of the environment (e.g., labs and farms, vs. animal shelters and zoos). Labs and precision farms place far greater research effort into monitoring acoustically active animals, to promote high animal health and welfare. In contrast, animal shelters and zoos have focused on environmental sound as a general putative stressor, whereas animals' responses to a specific sound property (such as decibel level or the level of anthrophony) are overlooked [e.g., (7), (78), (91)]. In all captive environments, there has been a clear reliance on logging decibels vs. taking sound recordings, and decibels have not always been collected at different frequencies to take the full features of sound into account. Sound mitigations and acoustic enrichment sometimes appear fairly *ad hoc*, but we fully appreciate the difficulties in making significant acoustic changes to enclosures, particularly those open to the public. It is evident there is no such thing as “standard” captive enclosure sound (or noise), due to large variations within and between these environments and the species housed. This being said, more standardized experimental design would help ascertain whether variations in sound derive from variation in methods. Our paper is a descriptive review of a very broad range of literature. It maintains a practical, methodological focus so that it can encourage more researchers to perform captive bioacoustic research. However, we also hope that our descriptive review may inspire systematic reviews or meta-analyses (144). For example, a systematic review to quantify the frequency and diversity of sound measurement/s across different captive settings and taxonomic groups would serve to strengthen our opinion that captive methods are disjointed. As stated earlier, we have excluded discussions of specific research findings (e.g., behavioral and welfare effects of sound) from our paper, but a future study assessing the value of various sound measurements for behavior and welfare assessment will also benefit the field. We now make three recommendations for acoustic research on captive animals.

### 5.1. Acoustic Monitoring in Zoos

We have shown that acoustic monitoring is mainly used in farm and laboratory environments (Section 3.2), but less commercial environments could certainly benefit from acoustic monitoring if they can find the funding and expertise. What might we specifically want to monitor in zoos and sanctuaries? Automatic call detection in zoos may have some value; for example, calls of vocal reptiles or amphibian species that are difficult to observe or occur at nighttime (145). Monitoring stress-related vocalization in a particular species has obvious benefits (146), particularly when animals cannot be observed reliably (e.g., overnight or during poor weather conditions). Another application of acoustic monitoring in captive environments could be *cue-counting*, i.e., counting the frequency of vocalizations or other animal sound per unit time and using this as a proxy for animal density or behavior. This could be used in larger zoo environments like safari parks with flocks of birds or herds of ungulates, where it is difficult to count animals by eye. The use of artificial intelligence systems to monitor sound in zoos feels ambitious at present, given the high computational power and initial human investment needed. But it may just be a matter of time; bioacoustics has now entered a “big data” era, shown by the emerging sub-field of computational bioacoustics (147, 148). To increase uptake in zoos, the novel hook is the conservation value of artificial intelligence; in other words, attracting the interest of conservation technologists who can study captive populations of threatened species. In fact, sound localization software in the field was recently modified for use in zoo enclosures; it works particularly well for loud and frequent vocalizing species with individual contact calls and a well-known vocal repertoire (149). Leading from this, there has been a small amount of wild research on *soundmarks*. A soundmark is a familiar sound that helps animals orient themselves within space (150). In captivity, soundmarks may be particularly static and predictable for animals. This leads to interesting research questions about the importance of soundmarks for captive animals, and whether they might impact reintroduction success.

### 5.2. Captive Soundscape Analyses

To our knowledge based on the published evidence, soundscape indices (for complexity, naturalness, and similarity) are not currently used in captive animal environments. The closest literature we can find examines the effect of farming activities on the natural soundscape but does not measure the farm soundscape *per se* (151). We believe the concept of soundscape ecology (i.e., the study of the effects of the soundscape on animals, [e.g., their physiological and behavioral responses; (134)] has real potential in captive settings, but to reach this potential it requires the use of indices, rather than just the use of the term “soundscape” (7, 106).

The value of different types of soundscape indices in captive environments is an interesting debate that will hopefully expand in the years to come as indices improve, particularly in their ability to discern biophony from geophony. Animal caregivers and researchers might be interested to quantify the natural biodiversity of zoo enclosures; in other words, how well they attract native birds, insects, and anurans as indicators of ecosystem health. Soundscape monitoring could also quantify the presence of pest species coming into captive enclosures (which vary greatly by region but may include cockroaches, birds, rodents, and rabbits) if these animals are highly cryptic and hard to detect visually. On balance, we think naturalness indices (Section 4.2.2) have the most value in captive environments. Zoos often strive to make enclosures as naturalistic as possible, but must also accommodate visitors and staff in a safe manner which justifies using typical construction materials. The NDSI may, therefore, help to detect whether human activities and the built environment are dominating the soundscape of zoo animals and whether this can be mitigated by introducing more sources of biophony through acoustic enrichment.

To date, no single index has been developed which can fully summarize a soundscape (136). And, similar to sound recording methods (Section 6.2), indices can be adversely affected by several factors, such as background noise (including geophony) and the distance between the sound source/s and the microphone. Soundscape ecology is still a relatively new and interdisciplinary field with rapid innovation, meaning there are no gold standard methods as yet which can be transferred from the field to captivity. Until then, we recommended using a combination of indices (136, 137), and aural monitoring is still important to reveal the identity of a sound that contributes to an index value. It has been suggested to record a minimum of 120 continuous hours from a site to derive reliable soundscape indices (137). This figure will be very aspirational for many projects; therefore, we recommend taking short sound recordings from several captive environments for comparison rather than one long measurement from one environment if this is more practical (100).

### 5.3. Measuring Sound From the Animal's Perspective

We recommend that going forwards, sound measurements (from both animals and the environment) are performed from the animal's “point of ear” as much as possible (4). This can be difficult for sensitive species, for example, nesting birds in a zoo (106), but circumvented to some degree by setting acoustic equipment up in advance and letting it measure sound automatically, rather than attempting to take measurements on the move. Taking sound measurements as close to where the animals reside in space as possible will take into account not only their location inside the enclosure (relative to different substrates) but also ear height [consider elephants vs. alligators, (108)] Sound measurement equipment should be set up within the species' hearing range (or ranges for a multi-species study). Interestingly, the soundscape concept in human audition considers how a listener perceives or understands sounds, but these subjective measures are currently overlooked in animal acoustics (152). For humans, subjective evaluation has included measuring levels of listener pleasure and what emotions or activities it may provoke. Work on animals will be more difficult, given that we cannot ask them directly how they feel, but could include focusing on short-term emotional responses to sound, the anticipation of different recurring sound events, and whether sound may provoke optimism or pessimism as demonstrated in humans (153). The influence of other stimuli (primarily visual) on soundscape perception has been explored in humans (154) and could be translated to animals.

## 6. A Practical Guide to Performing Acoustic Research on Captive Animals

### 6.1. Logging Sound Pressure Level

Decibel levels can be logged using a handheld or static logger. The former is useful when you are on the move and wish to take measurements sporadically at different locations, whereas the latter can be left in one location to record automatically. Loggers can be configured to take decibel levels at set time intervals (or responsively at the press of a button on the handheld version). It is possible to log raw readings or time-averaged values. Many loggers also come with sensors to simultaneously collect meta-data such as GPS coordinates and temperature. Careful configuration of the logger is critical before a logging session commences. Once the hearing capabilities of a species are known (Section 3.1.1), an appropriate *decibel weighting* can be chosen, which means a filter is applied on the logger to simulate the hearing range of that species. The Db(A) weighting is most commonly used for human and primate hearing ranges, whereas the Db(C) weighting is suitable for species with more sensitive hearing in the lower frequencies. A number of specific weightings have also been produced for marine mammals to suit their relatively very high-frequency hearing (154).

### 6.2. Recording Sound

**Figure 4:**
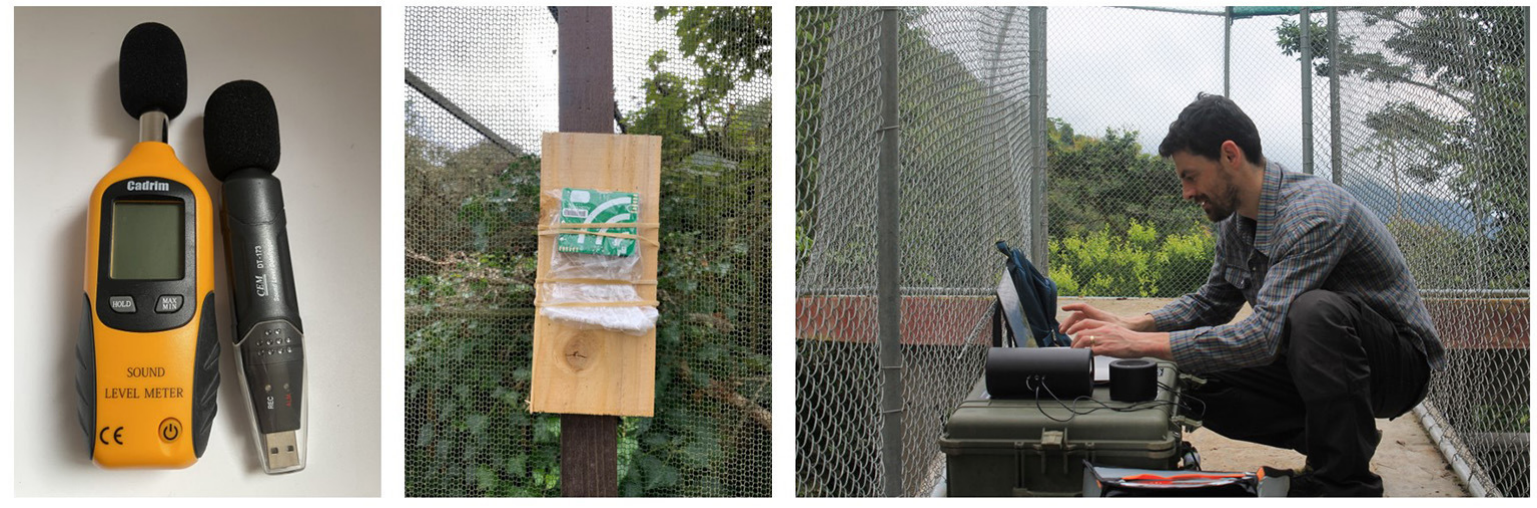
Equipment for acoustic research on captive animals. Left: handheld and static sound pressure level (decibel) loggers with foam windshields. Middle: an autonomous recording unit (Audiomoth) inside a waterproof sleeve, installed on a zoo enclosure to record the soundscape. Right: acoustic playback equipment consisting of a speaker and sound recorder pointed toward an enclosure. Photo credits: FE Clark, F Steinbrecher, JC Dunn.

A digital recording of a sound can be made using a handheld or static solid-state recorder (Figure 4). A passive sound recorder (also called an *autonomous recording unit*, ARU) records sound automatically according to pre-set instructions. One of the most popular ARUs for bioacoustics currently is the Audiomoth (155). ARUs are beneficial when your presence may disturb an animal (156), or when it is impractical to record sound on the move. Detailed discussions of ARU's can be found in (10) but, to summarize, current models tend to be low-cost, battery-powered, have open-source software, and record data to an SD card for later download. It is possible to pre-configure the sampling rate (the number of sound readings per unit time), periods of recording (e.g., during nighttime hours), or recording in response to a certain sound trigger (e.g., a particular animal vocalization) using an on-board detection algorithm (157). Some units will only record a specific sound; for example, echolocation click detectors record trains of clicks and thus the presence of at least one individual within range of the detector (158). It is also possible to set a logger to filter out unwanted sounds (155) such as human speech, which may be important for confidentiality reasons (159). Conservation technology is a burgeoning field, leading to the rapid development of ARUs (10) with compact size for animal-borne recording [see the μMoth, (160)] and low-cost underwater use [see the Hydromoth, (161)]. Many practical issues ARUs have experienced in the field (such as the absorbance of long-range radio signals by dense vegetation) are presumably less of an issue in captive environments because they are smaller and more hospitable. Whether you use an active or passive logger, the need for camouflage and weather-proofing will be highly context-specific.

We recommend recording uncompressed file formats (e.g.,.wav) rather than compressed file formats (e.g., MP3) because although uncompressed files take up more storage space, they do not lose quality from the original recording and therefore allow more fine-scale analyses. There are several considerations when recording: (i) the sampling frequency chosen will affect the range of frequencies recorded and vice versa; for example, when recording ultrasound, it is necessary to use a high sampling frequency and this also requires more storage and processing power; (ii) recordings must be taken above the *Nyquist frequency* (twice the highest frequency present in the sound) to avoid introducing artifacts into the recording; (iii) the bit depth of the recording is the number of possible amplitude values being recorded and is another important consideration. Increasing the bit depth will increase the resolution of the recording - 16 bits is standard, 24 bits is a better resolution but requires more storage space; (iv) background noise can cause a lot of interference with data quality (162, 163). Therefore, you should aim for a high signal-to-noise ratio; in other words, the amplitudes of the sound of interest and the background should be far apart so that the latter does not mask (cover up) the former. The signal-to-noise ratio can be increased using a parabolic reflector. Given the complexity of configuring acoustic loggers, it is always worth consulting with a bioacoustics expert, where possible, to review your specific needs. And in all cases, careful equipment maintenance, including weather protection, is vital to the success of sound recording.

To ensure ongoing data quality during research, it is usually recommended that researchers listen to recordings “live” through headphones and visually track their recordings via spectrograms (Section 2.4). Small, unintentional movements by the researcher or their equipment can introduce major sound artifacts into a recording that are difficult to remove post-production (33). Calibration of a sound logger is also important to ensure repeated accuracy of measurements. This involves playing a pure tone at a standardized amplitude and frequency, ideally in a sound-proof chamber (but in the real world in a very quiet room or using a calibrator that fits over the top of the microphone). Finally, you may be interested in measuring non-audible or solid-borne vibrations using a piezo-electronic accelerometer, which measures the acceleration of a surface and converts this to an electronic signal. An accelerometer can be mounted onto a substrate such as a window or a wall. When selecting an accelerometer, the frequency range of the accelerometer must cover the frequency range of interest.

Many sound recorders have in-built microphones which will work for some research requirements, but in general, a separate higher quality microphone should be used when recording animal sounds. First, a microphone (specialized microphones are required for ultrasound, infrasound, and underwater sound) is required to transduce acoustic signals into an electrical signal. A directional microphone is best when the sound of interest has a discrete source (e.g., a vocalizing animal), whereas general environmental or animal group sound is best captured using an omnidirectional microphone. A spatial array of (directional) microphones can be used to estimate the distance and directionality of a sound signal, and therefore used to infer the location of a vocalizing animal (59, 164–166). Microphones should be placed as close to the sound source as possible without direct interference, and away from vegetation or other substrates which may absorb or reflect sound. An additional personal microphone attached to your collar can be used to record field notes but be wary of introducing unintentional artifacts (sound generated by you or your equipment) into the recording. It is good practice to record “meta-data”, e.g., geographical location, temperature, humidity, water salinity, and air pressure/wind direction that may affect the sound recording. A windshield (a synthetic fur, foam, or mesh microphone cover) can block wind from hitting the microphone and therefore reduce the detection of unwanted sound. Alternatively, a microphone high-pass filter can be used to minimize low-frequency wind noise being detected on the recording. A microphone can be animal-borne (161 attached to a radio collar or ear tag that can transmit sound recordings to a receiver [e.g., farmed ungulates, (45); African elephants *Loxodonta Africana*, (49); primates, (167)]. Note that this constitutes invasive research because it involves animal capture, and therefore requires specific ethical approval (168).

### 6.3. Playing Sound

Creating acoustic playback stimuli can be challenging. For a “silence” or control condition, researchers may create an empty.wav file (at zero amplitude), play white noise, or use actual background noise with no additional playback recording. For a particular sound, several online sound repositories are available. Supplementary Table 1 provides some useful examples of these repositories, but is by no means an exhaustive list. However, it is important to exercise caution when using recordings made by others/for other reasons, because older acoustic equipment may not have recorded the full frequency spectrum of the sound, and most animal recordings lack context [a wide variety of factors are known to affect animal calls; (169)]. In some circumstances, it may be worth the time to record new sound stimuli using a standardized protocol which can then be reported alongside the findings. Scheduling playback experiments around routine cleaning and other husbandry sound is important so that playback stimuli are not competing with very intense sound levels (170).

Like microphones, speakers can be directional or omnidirectional, and this choice will depend on what sound stimuli you intend to broadcast (e.g., an animal vocalization vs. ambient background sound). Speakers can also be combined into spatial arrays to create more widespread sound production. No matter which speaker is chosen, it will be limited in its ability to reproduce all the properties of the sound (4). For this reason, you must consider whether using the “real” noise stimuli is feasible, rather than a playback version. For aquatic animals, a water-coupled speaker could be used; sound from a speaker in the air vibrates through a flexible water bladder which is placed onto a tank wall [e.g., fish, (171); marine turtles, *Caretta caretta*; (172)]. We advise that you pilot-test all playback equipment *in situ* (where the experiment will take place), but out of earshot of the test subjects, to avoid habituation.

### 6.4. Summarizing and Analyzing Sound

In most environments, sound is not constant and therefore sound pressure levels fluctuate over time. So, in many cases, you may want to summarize decibels collected over a particular period. For example, L_90_ specifies the decibel level that was exceeded in a recording for 90% of the time (L_50_ is the decibel level exceeded for 50% of the time). Time-averaged values are a good option to summarize chronic or frequent sound. For example, L_eq_ is a measure of average sound pressure level over a specified period, and is useful for constant ambient noise levels (e.g., an indoor enclosure with constant HVAC sound). L_max_ and L_min_ are the maximum and minimum sound pressure levels respectively over a given time period, and L_max_ is particularly useful for short, abrupt noise events (e.g., dog barks or bangs). A more mathematically intensive yet biologically valid way to summarize sound pressure level is to calculate the root-mean-square of decibel levels to represent an average pressure level over a given time. To summarize frequencies, average or peak frequencies are commonly used. With all of these measures, you should specify over what interval of time the measurements were made. A major drawback is these summary metrics do not tell us the biological significance of the sound. Furthermore, there is a lack of guidance on maximum noise thresholds for animals, so researchers often extrapolate from human thresholds (e.g., from the World Health Association and National Institute for Occupational Safety and Health).

Extracting sound metrics (e.g., the minimum, maximum, and average frequencies; the timing and duration of a specific sound of interest) from a decibel logging session or sound recording is a considerable research task in itself (40). In recent years, much of the manual labor involved in extracting and analyzing metrics from sound visualization graphs (Figure 3) has fortunately been superseded by bioacoustics software and artificial intelligence. A range of proprietary and open-source software is available including Raven Pro (173), Praat (174), Avisoft (175), and packages within R including SoundEcology (176). Further detail and recommendations on software can be found in (10). Software packages allow sound metrics to be extracted and downloaded in tabular form for further statistical analysis, such as correlations, ANOVA, linear and mixed-effects models, or discriminant function analysis (33, 56, 70). Artificial detection and classification of sound (i.e., detecting whether a particular sound and/or the type of sound, is present/absent in a recording) has burgeoned over the past decade, due to advancements in machine learning (177) and other statistical techniques (178). We recommend the R package SeeWave (179) for the calculation of soundscape indices.

### 6.5. Acoustic Monitoring Workflow

**Figure 5:**
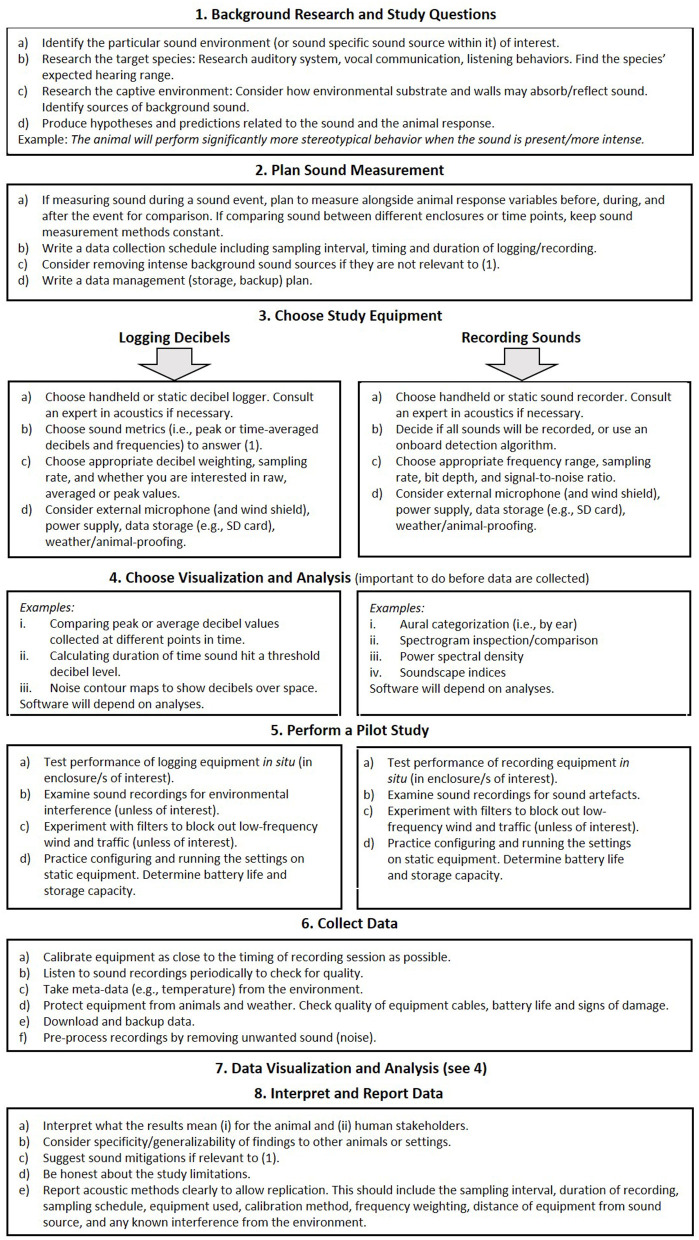
A practical workflow to monitor sound in captive environments.

We end with a suggested sound measurement workflow (Figure 5). This is intended for animal care staff and researchers interested in monitoring environmental sound: either background sound or sound events in their facility. This can include decibel logging and/or sound recordings. There is no guaranteed “gold standard” method because as our review demonstrates, sound measurement is heavily context-dependent. However, this workflow offers several prompts to measure and interpret sound in a meaningful way. It draws attention to frequently overlooked aspects of study design and execution, such as the need to go “beyond the decibel”. Here we cover the recording but not the generation of sound (i.e., no playback experiments, and no experimental control over sound sources).

## 7. Conclusions

Sound measurement in captive environments has many forms, from pure bioacoustics research to acoustic monitoring for animal welfare. However, there have been many disparate, disconnected approaches to the measurement of sound in captive environments, and progress may have been impeded by a lack of accessible guides to sound measurement. To keep pace with the growth of automated acoustics in field environments, captive environments must move away from simplistic decibel recordings and toward measurements of the full soundscape. When a researcher has a solid sound measurement protocol for their environment in their armory, they can use it to investigate the effect of sound on any dependent variable(s) of choice, or to measure sound as a potential nuisance variable. Where possible, we fully encourage collaboration with acoustic specialists. And to combat the replication crisis, acoustics methods should be fully described within publications or their Supplementary Material.

## Author Contributions

All authors listed have made a substantial, direct, and intellectual contribution to the work and approved it for publication.

## Conflict of Interest

The authors declare that the research was conducted in the absence of any commercial or financial relationships that could be construed as a potential conflict of interest.

## Publisher's Note

All claims expressed in this article are solely those of the authors and do not necessarily represent those of their affiliated organizations, or those of the publisher, the editors and the reviewers. Any product that may be evaluated in this article, or claim that may be made by its manufacturer, is not guaranteed or endorsed by the publisher.
