# Supplementary material for: From Soundwave to Soundscape: A Guide to Acoustic Research in Captive Animal Environments
Source: Front Vet Sci. 2022 Jun 16;9:889117. doi: 10.3389/fvets.2022.889117 (PMC9244380; doi:10.3389/fvets.2022.889117)
Supplement: Supplementary file 1 [file Table_1.DOCX]

**Supplementary Material**

Table 1. Sound archives for acoustic playback research on animals.

| **Library name and web address** | **Description** |
| --- | --- |
| Animal Sound Archive (Museum für Naturkunde Berlin)  <http://www.animalsoundarchive.org> | c. 120,000 nature recordings. Free for non-commercial use. Search in English, German, and by taxonomic name. |
| Australian National Wildlife Collection  <https://www.csiro.au/en/Research/Collections/ANWC/About-ANWC/Our-wildlife-sound-archive> | c. 60,000 recordings. Currently in the process of digitization. |
| BBC Sound Effects  <https://sound-effects.bbcrewind.co.uk/> | c. 30,000 sound effects including c. 17,000 nature sounds. Many are freely downloadable for non-commercial use. Others can be paid for. |
| Borror Laboratory of Bioacoustics (Ohio State University)  <https://blb.osu.edu> | c. 40,000 animal sounds. Freely available for listening online. |
| British Library of Sounds  <https://www.bl.uk/collection-guides/wildlife-and-environmental-sounds> | c. 240,000 sound and video recordings. Freely available for listening online. |
| The Fonozoo (National Museum of Natural Sciences of Madrid)  <http://www.fonozoo.com> | c. 11,000 sounds. Search in Spanish and English.  Recordings need to be requested, not all available directly online. |
| Remote Environmental Assessment Library <https://www.remoteenvironmentalassessmentlaboratory.com/> | Birdsong, amphibians, and soundscapes. Permission must be sought from the recording owner. |
| BioAcoustica <https://bio.acousti.ca/> | Animal sounds. Recordings are individually licensed. |
| Florida Museum Bioacoustic Archive | Mainly birds. Free to download. |
| Macaulay Library (Cornell Lab of Ornithology)  <https://www.macaulaylibrary.org> | c. 370,000 sounds and 60,000 videos. Request a sound and receive a link for non-commercial use. |
| Xeno-canto Bird Sounds  <https://xeno-canto.org/> | c. 400,000 sounds from 10,000 bird species. Free to download. Use with the WarbleR package in R. |
